# Prognostic Value of the Glasgow Prognostic Score on Overall Survival in patients with Advanced Non-Small Cell Lung Cancer

**DOI:** 10.7150/jca.52215

**Published:** 2021-03-01

**Authors:** Mingmei Pan, Yun Zhao, Jianbo He, Huanqiong Wu, Yujia Pan, Qitao Yu, Shaozhang Zhou

**Affiliations:** 1 College of Oncology, Guangxi Medical University, No.22 Shuangyong Road, 530021, Nanning City, Guangxi Zhuang Autonomous Region, China.; 2 Department of Respiratory Oncology, Guangxi Medical University Affiliated Tumor Hospital, No.71 Heti Road, 530021, Nanning City, Guangxi Zhuang Autonomous Region, China.

**Keywords:** Glasgow Prognostic Score, prediction, overall survival, non-small cell lung cancer

## Abstract

**Background:** Findings from previous studies regarding the association between the Glasgow Prognostic Score (GPS) and overall survival (OS) of patients with advanced non-small cell lung cancer (NSCLC) were limited. This study aimed to investigate the prognostic value of GPS in patients with advanced NSCLC after adjusting for potential confounding factors.

**Methods:** A retrospective cohort study was conducted in 494 patients with advanced NSCLC between 2009 and 2019. Clinicopathological characteristics (including GPS) were analyzed to determine predictors of OS using univariate and multivariate Cox proportional hazards models. Survival curves were estimated using the Kaplan-Meier method.

**Results:** Of the enrolled patients with advanced NSCLC, 66.46% were men and 53.85% were aged <60 years. The percentages of GPS scores of 0, 1, and 2 were 36.44%, 36.03%, and 27.53%, respectively. The median OS of the GPS 0, 1, and 2 groups were 23.27, 14.37, and 10.27 months, respectively (log-rank P <0.0001). A higher GPS was independently associated with an increased risk of death (P for trend = 0.0004) after full adjustment for potential confounders. The risk of death increased by 77% in the GPS 1 group (hazard ratio [HR]=1.77, 95% confidence interval [CI]=1.22-2.57, P=0.0027) and 109% in the GPS 2 group (HR=2.09, 95%CI=1.36-3.22, P=0.0008) compared with the GPS 0 group after adjustment. We did not find significant heterogeneity among the analyzed subgroups apart from sex (P interaction=0.017).

**Conclusion:** High pretreatment GPS is independently associated with worse OS in patients with advanced NSCLC. GPS should be considered in patient counseling and decision-making and needs to be further validated by large-cohort and prospective studies.

## Introduction

Lung cancer has the highest morbidity among all cancer types and is the leading cause of cancer-related deaths worldwide, of which non-small cell lung cancer (NSCLC) accounts for 85% 1. Although diagnostic technologies and therapeutic strategies for NSCLC have markedly improved, the prognosis of patients with advanced NSCLC remains poor, with a five-year survival rate of < 5%.

Traditionally, the tumor, node, and metastasis (TNM) staging system as a predictive tool for patients with lung cancer is most used in clinical practice. However, the TNM staging system alone is far from perfect to evaluate prognostic outcomes because cancer survival may be influenced by tumor characteristics as well as host situations 2-4. It fails in further classifying advanced NSCLC so that different outcomes can be observed in patients with the same TNM stage. What is important, however, is that the understanding of advanced NSCLC survival may assist clinicians during treatment planning and decision-making. Therefore, reliable predictive factors for individualized prediction of survival in NSCLC are urgently required.

To date, several studies have verified that systemic inflammation plays a significant role in the development and progression of a variety of cancers 5-8. Inflammation-based prognostic scores, including systematic inflammation-immune score and platelet-to-lymphocyte ratio, have been proved to have prognostic value in patients with cancer. There is growing recognition that the decline in nutritional status is associated with a worse prognosis. As a combination of albumin and C-reactive protein (CRP), Glasgow Prognostic Score (GPS) reflects not only systemic inflammation but also nutritional status. GPS can be obtained during routine in-patient blood sampling, which is simple to measure, inexpensive, repeatable, and well standardized. Research has shown that GPS is independently associated with survival in many cancers, including metastatic colorectal cancer, NSCLC, colon cancer, and gastric cancer 9-12. Although some studies adjusted for variables such as age, sex, pathological type, smoking history, treatment plan, and others, some variables that have been confirmed to have an impact on the prognosis of NSCLC such as liver metastasis, the sum of treatment lines, gene mutation status, and others, were not included in the adjustment. Therefore, this study aimed to investigate whether GPS is independently associated with overall survival (OS) in patients with advanced NSCLC after adjusting for the above variables.

## Materials and methods

### Study population

Patients with advanced NSCLC who were hospitalized at the Guangxi Medical University Affiliated Tumor Hospital, Guangxi Province city, China between January 2009 and May 2019, were enrolled. All patients were strictly followed up until August 2019. The inclusion criteria were: (1) patients with cytologically or pathologically confirmed NSCLC; (2) patients were classified as having TNM stage IIIB or IV according to the seventh edition American Joint Commission on Cancer guidelines (AJCC V7.0); (3) patients with baseline albumin, CRP, and complete follow-up data. The exclusion criteria were: (1) patients with a history of other malignancy; (2) patients with chronic diseases (e.g., hepatitis, cholecystitis) that could interfere with the measured laboratory parameters; (3) OS of < 90 days. According to the inclusion and exclusion criteria, a total of 494 patients were included in the study.

### Evaluated variables

GPS, as categorical variables, were obtained at baseline. Hypoalbuminemia (<35 g/L) and CRP elevation (>10 mg/L) were each given a score of 1; thus, the GPS was calculated as 0, 1, or 2 13. OS was defined as the period from the diagnosis of NSCLC to death or the last follow-up. Covariates obtained at baseline included four aspects: (1) demographic data: sex, age, and smoking history; (2) tumor characteristics: pathology, epidermal growth factor receptor (EGFR) mutation status, liver metastasis, the sum of metastatic sites, and TNM stage; (3) host situation: the Eastern Cooperative Oncology Group Performance Status (ECOG PS), plasma D-dimer levels, white blood cell (WBC) and lactate dehydrogenase (LDH) levels; and (4) treatment situation: treatment and the sum of treatment lines.

Tumor histology was classified according to the World Health Organization (WHO) classification of tumors (third edition). The AJCC V7.0 was used to determine tumor stages. We used computed tomography combined with Response Evaluation Criteria in Solid Tumors version 1.1 to assess the treatment efficacy every two cycles until treatment cessation or disease progression 14. The combination therapies in this study were defined as chemotherapy combined with targeted therapy, anti-angiogenic therapy, or immunotherapy. The entire process of data collection was non-selective and consecutive. Data were stored in an electronic data acquisition system. Our data did not include identifiable patient data to safeguard patients' privacy. Informed consent was not required because this was a retrospective cohort study. The study was approved by the Ethics Committee of Guangxi Medical University Affiliated Tumor Hospital.

### Statistical analyses

Continuous variables were converted to categorical variables based on routine cutoff points in clinical applications. Baseline characteristics are presented as proportions for categorical variables. We used the chi-square test (χ^2^) to test for differences between different groups. Fisher's exact test was used to compare the variables when the number of samples was ≤ 5. Kruskal-Wallis H test was applied to test between multiple groups presenting as rank variables. Univariable Cox regression was used to analyze the effect of several confounding risk factors on survival and Cox proportional hazards models were applied to evaluate the prognostic value of GPS after adjustment for other confounding factors. We adjusted variables in Cox proportional hazards models when the hazard ratio (HR) changed by at least 10% after a variable was adjusted or P-value was ≤ 0.05 in the univariable analysis. We converted the GPS into a continuous variable and calculated the P for trend. Subgroup analyses were performed using stratified Cox proportional hazard models, and an interaction test was performed. Survival curves were constructed using the Kaplan-Meier method with the log-rank test. All analyses were performed using the statistical software packages R (http://www.R-project.org, The R Foundation) and EmpowerStats (http://www.empowerstats.com, X&Y Solutions, Inc., Boston, MA). P-values ≤ 0.05 (two-sided) were considered statistically significant.

## Results

### Baseline characteristics of patients with advanced NSCLC

A total of 494 patients with advanced NSCLC were selected for the final data analysis by strict screening criteria as previously described. During the follow-up period, 316 (63.97%) patients died. The baseline characteristics of these selected patients stratified according to GPS are shown in Table 1. Of the 494 patients with advanced NSCLC, 66.40% were men, 53.85% were aged ≤ 60 years, 50.81% were non-smokers, 88.65% had an ECOG PS of 0 or 1, 74.70% had adenocarcinoma and 84.82% were diagnosed as having TNM stage IV. The percentages of GPS scores of 0, 1, and 2 were 36.44%, 36.03%, and 27.53%, respectively. A total of 49.19% of patients were tested for the EGFR gene, and 41.98% of them had EGFR mutation. Among the patients with GPS 2, the proportion of patients aged ≥ 60 years, ECOG PS ≥ 2, squamous cell carcinoma, and high levels of LDH and WBC were higher than those in the other groups.

### Univariates analysis of OS

The results of the univariate analyses of OS are listed in Table 2. Sex, liver metastasis, other pathology types, the sum of metastatic organs, TNM stage, LDH level, and treatment were not associated with OS in the univariate analyses (P >0.05). We also found that EGFR mutation (HR=0.64, 95% confidence interval [CI]= 0.45-0.90) and treatment lines ≥2 (HR=0.56, 95% CI=0.43-0.72) were negatively associated with an increased risk of death. In contrast, univariate analysis showed that age ≥60 years, smoking, squamous cell lung carcinoma, ECOG PS ≥2, WBC ≥10×10^9^ /L, D-dimer level ≥0.5 mg/L, and GPS 1 and 2 were positively correlated with an increased risk of death in patients with advanced NSCLC (all P-values <0.05).

### Results of Cox proportional hazard model and Kaplan-Meier curves

As shown in Table 3, all models showed an increased risk of death in a GPS of 1 and 2 compared with a GPS of 0. The results remained statistically significant even after adjusting for potential confounders. In the fully adjusted model (adjusted for ECOG PS, TNM stage, and other covariates listed in Table 1), the risk of death increased by 77% in a GPS of 1 (HR=1.77, 95% CI=1.22-2.57) and 109% in a GPS of 2 (HR=2.09, 95% CI=1.36-3.22) compared with a GPS of 0. According to the trend test, higher GPS (1 and 2) was independently associated with an increased risk of death in patients with advanced NSCLC after adjustment for potential confounders (P for trend = 0.0004).

**Figure 1:**
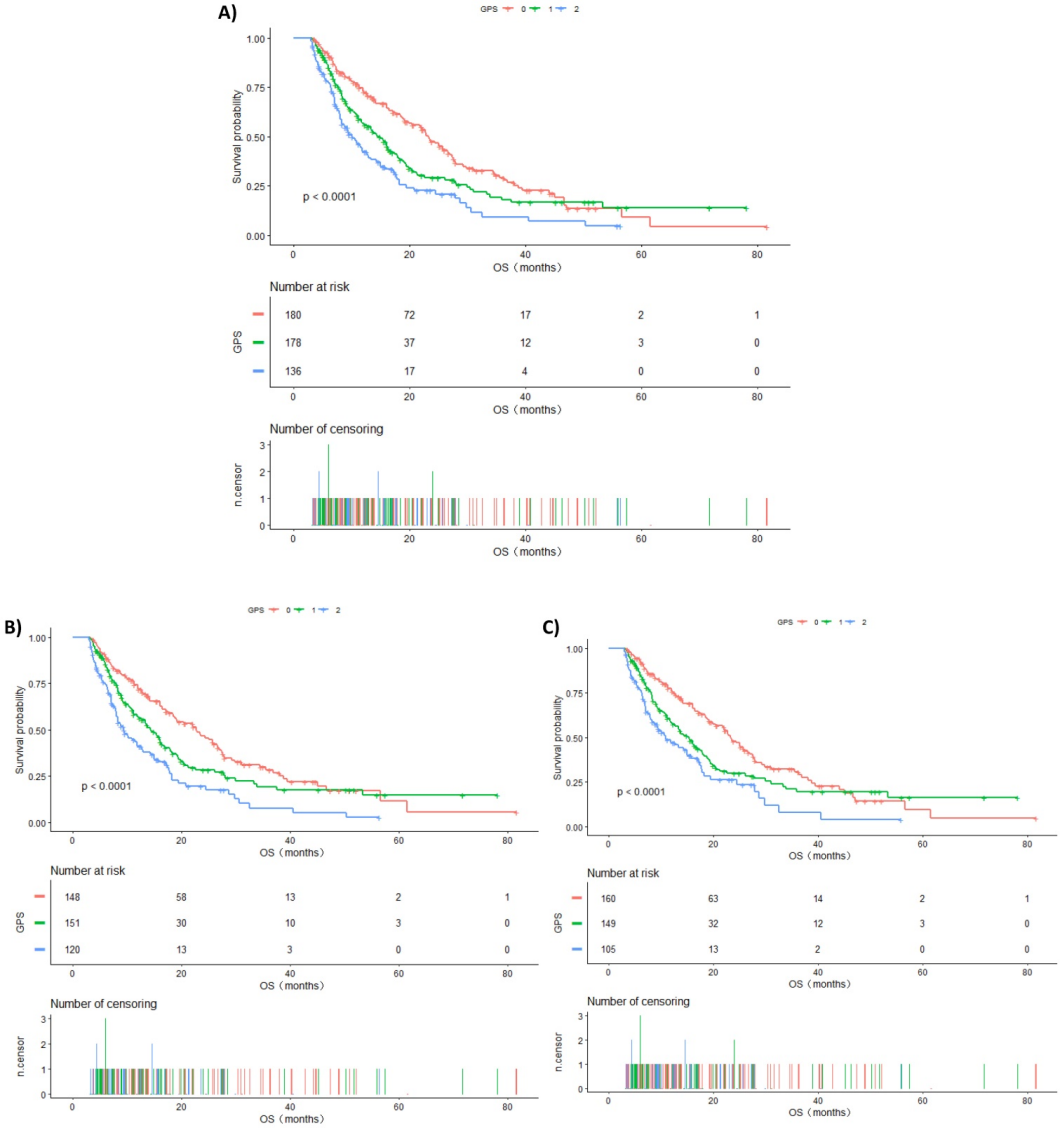
** A.** Kaplan-Meier curves of overall survival in all patients stratified by GPS. **B.** Kaplan-Meier curves of overall survival in patients having TNM IV stage stratified by GPS. **C.** Kaplan-Meier curves of overall survival in patients with ECOG PS 0-1 stratified by GPS.

The Kaplan-Meier curve for OS is shown in Figure 1. During the follow-up period, 316 (63.97%) patients died. Among all included populations, the median OS of GPS 0, 1, and 2 groups were 23.27, 14.37, and 10.27 months, respectively, and the difference between them was statistically significant (log-rank P <0.0001) (Figure 1A).

### Subgroup analysis of GPS and the risk of death in patients with advanced NSCLC after stratification by potential confounding covariates

**Figure 2:**
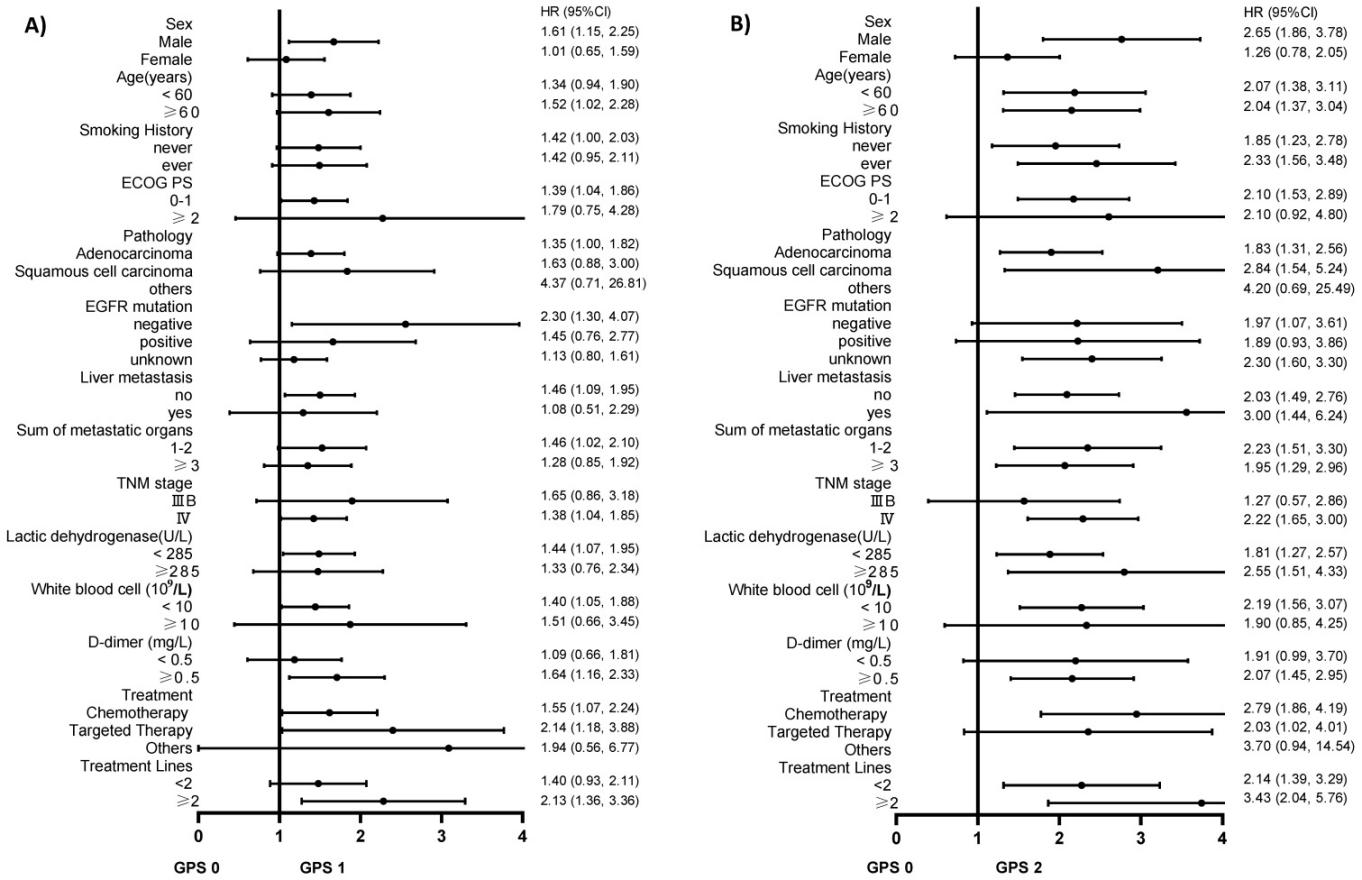
** A.** The association between GPS 1 and the risk of death compared to GPS 0 in various subgroups in the present advanced NSCLC study. **B.** The association between GPS 2 and the risk of death compared to GPS 0 in various subgroups in the present advanced NSCLC study.

We used the variables listed in Table 1 as the stratification variables to observe the trend of effect sizes (Table 4). The HRs of GPS 1 and 2 tended to increase in all subgroups compared with GPS 0. We did not observe significant heterogeneity among the analyzed subgroups apart from sex (P interaction=0.017). GPS was more strongly associated with an increased risk of death among men than among women. We used forest plots to present the HRs and their 95% CIs in Figure 2A and Figure 2B.

## Discussion

The prognosis of patients with advanced NSCLC remains unsatisfactory. In recent years, many researchers have actively sought potential reliable predictors to stratify cancer patients with a high risk of poor prognosis. Blood-based biomarkers have gradually become the research objects of researchers because of their economic and convenient features. Our findings identified that higher pretreatment GPS is significantly associated with an increased risk of death in patients with advanced NSCLC after fully adjusting for potential confounding covariates, indicating that GPS can serve as a predictor as well as a prognostic index beyond the TNM stage system for clinical decision-making. Patients with higher GPS may benefit from closer follow-up or earlier involvement in palliative care.

The prognostic value of CRP and albumin have been well established in a variety of cancers 15-18. However, their mechanisms have not been fully elucidated. Studies have shown that the inflammatory response can cause increased protein breakdown and a gradual decrease in nutrition because it has a direct catabolic effect on skeletal muscle, which leads to a decrease in treatment tolerance and survival in patients with cancer 19. Elinav et al. suggested that in cancer, the systemic inflammatory response may be secondary to tumor hypoxia, resulting in an imbalance of immune response, promoting tumor progression 20. Meanwhile, GPS is also a predictor of platinum-related toxicity, which may be associated with the decline of hepatic cytochrome P450 3A function under systemic inflammation 21, 22. These results remind us to pay attention to the treatment of systemic inflammatory response and malnutrition while treating tumors, which may markedly improve the prognosis of patients with cancer.

Heng Fan et al. suggested that GPS was a powerful prognostic factor in predicting survival in patients with operable and inoperable NSCLC in a sample of 2988 patients. Their multivariate survival analysis showed that higher GPS was associated with the risk of death in patients with operable NSCLC (HR= 2.228, 95%CI=1.447-3.431) and patients with inoperable NSCLC (HR=1.872, 95%CI=1.504-2.330) after adjusting for age, sex, TNM stage, and treatment 23. Similar findings were also obtained from other cancer researches such as gastric carcinoma and breast cancer 24-26. Their results are consistent with our findings. Nevertheless, LDH, D-dimer, EGFR mutation status, liver metastasis, and treatment lines, which were confirmed to exert their influence on the clinical outcomes in the previous studies, were not included for further covariate adjustment in their studies 27-30. In our study, 49.19% of patients were tested for the EGFR gene, and 41.98% of them had EGFR mutation. Targeted therapy approved as a standard first-line treatment for patients with lung cancer having genetic mutations and tyrosine kinase inhibitors have provided more opportunities for individualized treatment of these patients. The risk of death in patients with wild-type EGFR, liver metastasis, high LDH, D-dimer levels, and treatment lines < 2 tended to increase according to our univariate analysis. Not all trends were statistically significant, which may be due to the insufficient sample size. It is worth noting that the predictive effect of GPS on advanced NSCLC was still significant after adjusting for the above confounders in our study.

Subgroup analysis will help us in better understanding the trend of the risk of death in different populations. The results of this study found a stronger association in males than in females, although the mechanism is still unclear. Furthermore, ECOG PS is one of the key factors that we consider while developing treatment regimens for patients with advanced lung cancer. Interestingly, we found that patients with the same ECOG PS had an increased risk of death in the higher GPS group, and a statistically significant difference was observed in the ECOG PS 0-1 group (P for trend <0.0001). Similarly, the risk of death increased with the increase in GPS in the TNM IV stage group, and the difference was statistically significant (P for trend <0.0001). Kaplan-Meier survival curves were also performed to evaluate the OS of these subgroups (Figure 1B and Figure 1C). This demonstrated that GPS could further stratify these patients to consider whether they should undergo additional treatment or closer follow-up.

Our study has some strengths: (1) compared with many studies, we adjusted for more potential confounders and obtained the independent predictive effect of GPS on the prognosis of advanced NSCLC; (2) this study is a retrospective study and therefore susceptible to latent confounding factors. To minimize residual confounders, we used strict statistical adjustment; (3) we handled the target-independent variable as both continuous and categorical variables. Such an approach can enhance the robustness of results and reduce the contingency in the data analysis; (4) our findings should be useful in future research for the establishment of predictive models of lung cancer survival.

Although we adjusted for potential confounders, there is a possibility of residual or unknown confounding. Our research subjects were patients with advanced NSCLC, therefore, there is a certain inadequacy in the universality and extrapolation of research. In addition, according to the exclusion criteria, some patients were excluded from this study so that our results cannot be used for those people. Finally, a potential selection bias cannot be excluded because of the inherent limitations of a retrospective, single-center investigation.

In summary, in our study, we have confirmed that the pretreatment GPS can serve as a predictor of survival in patients with advanced NSCLC and should be considered in patient counseling and decision-making for additional therapy, which needs to be further validated by large-scale cohort and prospective studies.

## Figures and Tables

**Table 1:** Baseline characteristics of advanced NSCLC patients

	Total	GPS 0	GPS 1	GPS 2	P
	n=494 (100%)	n=180 (36.44%)	n=178 (36.03%)	n=136 (27.53%)	
**Sex**					0.028
male	328 (66.40)	106 (58.89)	126 (70.79)	96 (70.59)	
female	166 (33.60)	74 (41.11)	52 (29.21)	40 (29.41)	
**Age (years)**					<0.001
<60	266 (53.85)	111 (61.67)	100 (56.18)	55 (40.44)	
≥60	228 (46.15)	69 (38.33)	78 (43.82)	81 (59.56)	
**Smoking history**					0.025
never	250 (50.81)	104 (58.43)	87 (48.88)	59 (43.38)	
ever	242 (49.19)	74 (41.57)	91 (51.12)	77 (56.62)	
**ECOG PS^a^**					0.033
0-1	414 (88.65)	160 (93.02)	149 (88.17)	105 (83.33)	
≥2	53 (11.35)	12 (6.98)	20 (11.83)	21 (16.67)	
**Pathology**					0.023
Adenocarcinoma	369 (74.70)	142 (78.89)	139 (78.09)	88 (64.71)	
SCC^b^	109 (22.06)	31 (17.22)	35 (19.66)	43 (31.62)	
others	16 (3.24)	7 (3.89)	4 (2.25)	5 (3.68)	
**EGFR mutation^c^**					0.03
negative	141 (28.54)	39 (21.67)	58 (32.58)	44 (32.35)	
positive	102 (20.65)	48 (26.67)	34 (19.10)	20 (14.71)	
unknown	251 (50.81)	93 (51.67)	86 (48.31)	72 (52.94)	
**Liver metastasis**					0.817
no	419 (86.75)	154 (87.50)	150 (87.21)	115 (85.19)	
yes	64 (13.25)	22 (12.50)	22 (12.79)	20 (14.81)	
**Sum of metastatic organs**					0.157
1~2	267 (55.28)	107 (60.80)	92 (53.49)	68 (50.37)	
≥3	216 (44.72)	69 (39.20)	80 (46.51)	67 (49.63)	
**TNM stage^d^**					0.337
IIIB	75 (15.18)	32 (17.78)	27 (15.17)	16 (11.76)	
IV	419 (84.82)	148 (82.22)	151 (84.83)	120 (88.24)	
**Lactic dehydrogenase (U/L)**					<0.001
<285	358 (72.47)	146 (81.11)	133 (74.72)	79 (58.09)	
≥285	136 (27.53)	34 (18.89)	45 (25.28)	57 (41.91)	
**White blood cell (10^9^/L)**					<0.001
<10	374 (76.80)	166 (92.74)	129 (74.14)	79 (58.96)	
≥10	113 (23.20)	13 (7.26)	45 (25.86)	55 (41.04)	
**D-dimer (mg/L)**					<0.001
<0.5	134 (29.71)	64 (39.51)	49 (29.70)	21 (16.94)	
≥0.5	317 (70.29)	98 (60.49)	116 (70.30)	103 (83.06)	
**First-line treatment**					0.056
chemotherapy	243 (61.83)	77 (53.10)	97 (67.36)	69 (66.35)	
targeted therapy	111 (28.24)	53 (36.55)	35 (24.31)	23 (22.12)	
combination therapy	39 (9.92)	15 (10.34)	12 (8.33)	12 (11.54)	
**Treatment lines**					0.087
<2	199 (49.50)	71 (47.65)	66 (44.90)	62 (58.49)	
≥2	203 (50.50)	78 (52.35)	81 (55.10)	44 (41.51)	

**Table 2:** Univariates analysis of overall survival

	Statistics	Survival	P value
	n=494 (100%)	HR (95%CI)	
**Sex**			
male	328 (66.40)	1	
female	166 (33.60)	0.84 (0.67, 1.07)	0.158
**Age (years)**			
<60	266 (53.85)	1	
≥60	228 (46.15)	1.37 (1.10, 1.71)	0.0054
**Smoking history**			
never	250 (50.81)	1	
ever	242 (49.19)	1.31 (1.05, 1.63)	0.0175
**ECOG PS^a^**			
0-1	414 (88.65)	1	
≥2	53 (11.35)	1.43 (1.03, 2.00)	0.0345
**Pathology**			
Adenocarcinoma	369 (74.70)	1	
SCC^b^	109 (22.06)	1.57 (1.21, 2.04)	0.0008
others	16 (3.24)	1.41 (0.80, 2.46)	0.2324
**EGFR mutation^c^**			
negative	141 (28.54)	1	
positive	102 (20.65)	0.63 (0.45, 0.90)	0.0099
unknown	251 (50.81)	1.06 (0.81, 1.37)	0.6734
**Liver metastasis**			
no	419 (86.75)	1	
yes	64 (13.25)	1.23 (0.89, 1.69)	0.2034
**Sum of metastatic organs**			
<3	267 (55.28)	1	
≥3	216 (44.72)	1.10 (0.88, 1.38)	0.3964
**TNM stage^d^**			
IIIB	75 (15.18)	1	
IV	419 (84.82)	1.21 (0.88, 1.65)	0.243
**Lactic dehydrogenase (U/L)**			
<285	358 (72.47)	1	
≥285	136 (27.53)	1.20 (0.94, 1.53)	0.1368
**White blood cell (10^9^/L)**			
<10	374 (76.80)	1	
≥10	113 (23.20)	1.34 (1.04, 1.74)	0.0247
**D-dimer (mg/L)**			
<0.5	134 (29.71)	1	
≥0.5	317 (70.29)	1.40 (1.07, 1.82)	0.0146
**GPS**			
0	180 (36.44)	1	
1	178 (36.03)	1.41 (1.08, 1.83)	0.0116
2	136 (27.53)	2.10 (1.59, 2.78)	<0.0001
**First-line treatment**			
chemotherapy	243 (61.83)	1	
targeted therapy	111 (28.24)	0.81 (0.60, 1.09)	0.1638
combination therapy	39 (9.92)	0.70 (0.42, 1.17)	0.1762
**Treatment lines**			
<2	199 (49.50)	1	
≥2	203 (50.50)	0.56 (0.43, 0.72)	<0.0001

**Table 3:** Overall survival results of unadjusted and adjusted Cox proportional hazard model

GPS	N	With outcome numbers	Crude	Adjusted Model
			HR (95%CI) P value	HR (95%CI) P value
0	180	109	1	1
1	178	113	1.41 (1.08, 1.83) 0.0116	1.77 (1.22, 2.57) 0.0027
2	136	94	2.10 (1.59, 2.78) <0.0001	2.09 (1.36, 3.22) 0.0008
**P for trend**			<0.0001	0.0004

Adjusted Model adjust for: Sex, Age, Smoking history, Eastern Cooperative Oncology Group performance status, Pathology, Liver metastasis, Sum of metastatic organs, TNM stage, Epidermal Growth Factor Receptor, Lactic dehydrogenase, White blood cell, D-Dimer, Treatment lines, Treatment.

**Table 4:** Subgroup analysis of GPS and the risk of death in patients with NSCLC after stratified by potential confounders

	N	GPS 0	GPS 1	GPS 2	P for trend	P interaction
			HR (95%CI)	HR (95%CI)		
**Sex**						0.017
male	328	1	1.61 (1.15, 2.25)	2.65 (1.86, 3.78)	<0.0001	
female	166	1	1.01 (0.65, 1.59)	1.26 (0.78, 2.05)	0.3984	
**Age (years)**						0.802
<60	266	1	1.34 (0.94, 1.90)	2.07 (1.38, 3.11)	0.0006	
≥60	228	1	1.52 (1.02, 2.28)	2.04 (1.37, 3.04)	0.0005	
**Smoking history**						0.4351
never	250	1	1.42 (1.00, 2.03)	1.85 (1.23, 2.78)	0.0023	
ever	242	1	1.42 (0.95, 2.11)	2.33 (1.56, 3.48)	<0.0001	
**ECOG PS^a^**						0.7226
0-1	414	1	1.39 (1.04, 1.86)	2.10 (1.53, 2.89)	<0.0001	
≥2	53	1	1.79 (0.75, 4.28)	2.10 (0.92, 4.80)	0.082	
**Pathology**						0.729
Adenocarcinoma	369	1	1.35 (1.00, 1.82)	1.83 (1.31, 2.56)	0.0004	
SCC^b^	109	1	1.63 (0.88, 3.00)	2.84 (1.54, 5.24)	0.0008	
others	16	1	4.37 (0.71, 26.81	4.20 (0.69, 25.49	0.1145	
**EGFR mutation^c^**						0.0649
negative	141	1	2.30 (1.30, 4.07)	1.97 (1.07, 3.61)	0.0311	
positive	102	1	1.45 (0.76, 2.77)	1.89 (0.93, 3.86)	0.0681	
unknown	251	1	1.13 (0.80, 1.61)	2.30 (1.60, 3.30)	<0.0001	
**Liver metastasis**						0.208
No	419	1	1.46 (1.09, 1.95)	2.03 (1.49, 2.76)	<0.0001	
Yes	64	1	1.08 (0.51, 2.29)	3.00 (1.44, 6.24)	0.0062	
**Sum of metastatic organs**						0.8993
1-2	267	1	1.46 (1.02, 2.10)	2.23 (1.51, 3.30)	<0.0001	
≥3	216	1	1.28 (0.85, 1.92)	1.95 (1.29, 2.96)	0.0017	
**TNM stage^d^**						0.2811
IIIB	75	1	1.65 (0.86, 3.18)	1.27 (0.57, 2.86)	0.3646	
IV	419	1	1.38 (1.04, 1.85)	2.22 (1.65, 3.00)	<0.0001	
**Lactic dehydrogenase (U/L)**						0.1996
<285	358	1	1.44 (1.07, 1.95)	1.81 (1.27, 2.57)	0.0005	
≥285	136	1	1.33 (0.76, 2.34)	2.55 (1.51, 4.33)	0.0003	
**White blood cell (10^9^/L)**						0.9672
<10	374	1	1.40 (1.05, 1.88)	2.19 (1.56, 3.07)	<0.0001	
≥10	113	1	1.51 (0.66, 3.45)	1.90 (0.85, 4.25)	0.0964	
**D-dimer (mg/L)**						0.4186
<0.5	134	1	1.09 (0.66, 1.81)	1.91 (0.99, 3.70)	0.106	
≥0.5	317	1	1.64 (1.16, 2.33)	2.07 (1.45, 2.95)	<0.0001	
**First-line treatment**						0.5202
chemotherapy	243	1	1.55 (1.07, 2.24)	2.79 (1.86, 4.19)	<0.0001	
targeted therapy	111	1	2.14 (1.18, 3.88)	2.03 (1.02, 4.01)	0.0157	
combination therapy	39	1	1.94 (0.56, 6.77)	3.70 (0.94, 14.54)	0.0597	
**Treatment Lines**						0.4744
<2	199	1	1.40 (0.93, 2.11)	2.14 (1.39, 3.29)	0.0007	
≥2	203	1	2.13 (1.36, 3.36)	3.43 (2.04, 5.76)	<0.0001	

**Abbreviations:** ^a^Eastern Cooperative Oncology Group performance status; ^b^Squamous cell carcinoma; ^c^Epidermal Growth Factor Receptor mutation; ^d^Tumor Node Metastasis stage.

## Abbreviations

NSCLC: non-small cell lung cancer
OS: overall survival
GPS: Glasgow Prognostic Score
CI: confidence interval
HR: hazard ratio
TNM staging system: the tumor, node, and metastasis staging system
CRP: C-reactive protein
AJCC V7.0: the seventh edition American Joint Commission on Cancer guidelines
EGFR: epidermal growth factor receptor
ECOG PS: Eastern Cooperative Oncology Group Performance Status
WBC: white blood cell
LDH: lactate dehydrogenase
WHO: World Health Organization
SCC: Squamous cell carcinoma
